# Learning about COVID-19: sources of information, public trust, and contact tracing during the pandemic

**DOI:** 10.1186/s12889-022-13731-7

**Published:** 2022-07-15

**Authors:** Philip S. Amara, Jodyn E. Platt, Minakshi Raj, Paige Nong

**Affiliations:** 1 Division of Learning and Knowledge Systems, Department of Learning Health Sciences (DLHS), University of Michigan Medical School, 1161A 300 N. Ingalls Building, 11th Floor, Ann Arbor, MI 48109-5403 USA; 2 Division of Learning and Knowledge Systems, Department of Learning Health Sciences, University of Michigan Medical School, 300 N. Ingalls - 1161 NIB – 5403, Ann Arbor, MI 48109-5403 USA; 3 Department of Kinesiology and Community Health, College of Applied Health Sciences, University of Illinois at Urbana-Champaign, 2007 Huff Hall, 1206 South Fourth Street, Champaign, IL 61820 USA; 4 Department of Health Management and Policy, School of Public Health, University of Michigan, Ann Arbor, MI 48109-5403 USA

**Keywords:** Contact tracing, COVID-19, Information sources, Misinformation, Public trust

## Abstract

**Objective:**

To assess the association between public attitudes, beliefs, and information seeking about the COVID-19 pandemic and willingness to participate in contact tracing in Michigan.

**Methods:**

Using data from the quarterly Michigan State of the State survey conducted in May 2020 (*n* = 1000), we conducted multiple regression analyses to identify factors associated with willingness to participate in COVID-19 contact tracing efforts.

**Results:**

Perceived threat of the pandemic to personal health (*B* = 0.59, *p* = <.00, Ref = No threat) and general trust in the health system (*B* = 0.17, *p* < 0.001), were the strongest positive predictors of willingness to participate in contact tracing. Concern about misinformation was also positively associated with willingness to participate in contact tracing (*B* = 0.30, *p* < 0.001; Ref = No concern). Trust in information from public health institutions was positively associated with willingness to participate in contact tracing, although these institutions were not necessarily the main sources of information about COVID-19.

**Conclusion:**

Policy makers can enhance willingness to participate in public health efforts such as contact tracing during infectious disease outbreaks by helping the public appreciate the seriousness of the public health threat and communicating trustworthy information through accessible channels.

## Introduction

At the end of 2020, the severe acute respiratory syndrome-coronavirus 2 (SARS-CoV-2; or COVID-19) pandemic had been linked to more than 19.3 million confirmed cases in the United States including over 377,000 deaths [1]. Michigan was designated a hotspot in the first months of the pandemic [2]. Prior to the availability of an effective vaccine, other public health interventions like mask wearing, social distancing, isolation, quarantine, and contact tracing were primarily used to control the pandemic [3, 4]. Contact tracing, a long-standing public health tool, involves identifying the network of individuals that encounter an infected person and screening them for symptoms of the disease. Contacts with symptoms are quarantined and monitored during the incubation period of the disease. If positive when tested, they are treated and their contacts are identified, to prevent further spread of the disease [5].

At the outset of the COVID-19 pandemic, contact tracing was a critical component of prevention efforts, but its traditional modalities of being conducted in-person or by phone were challenging given the spread of COVID-19 as an airborne illness. This led to the development of digital contact tracing tools including outbreak response, proximity tracking and symptom tracking tools [6]. During COVID-19, state public health departments and private companies such as Google and Apple explored the use of proximity tracking tools such as health apps and geo-location technologies to trace possible contacts [7].

**Fig. 1:**
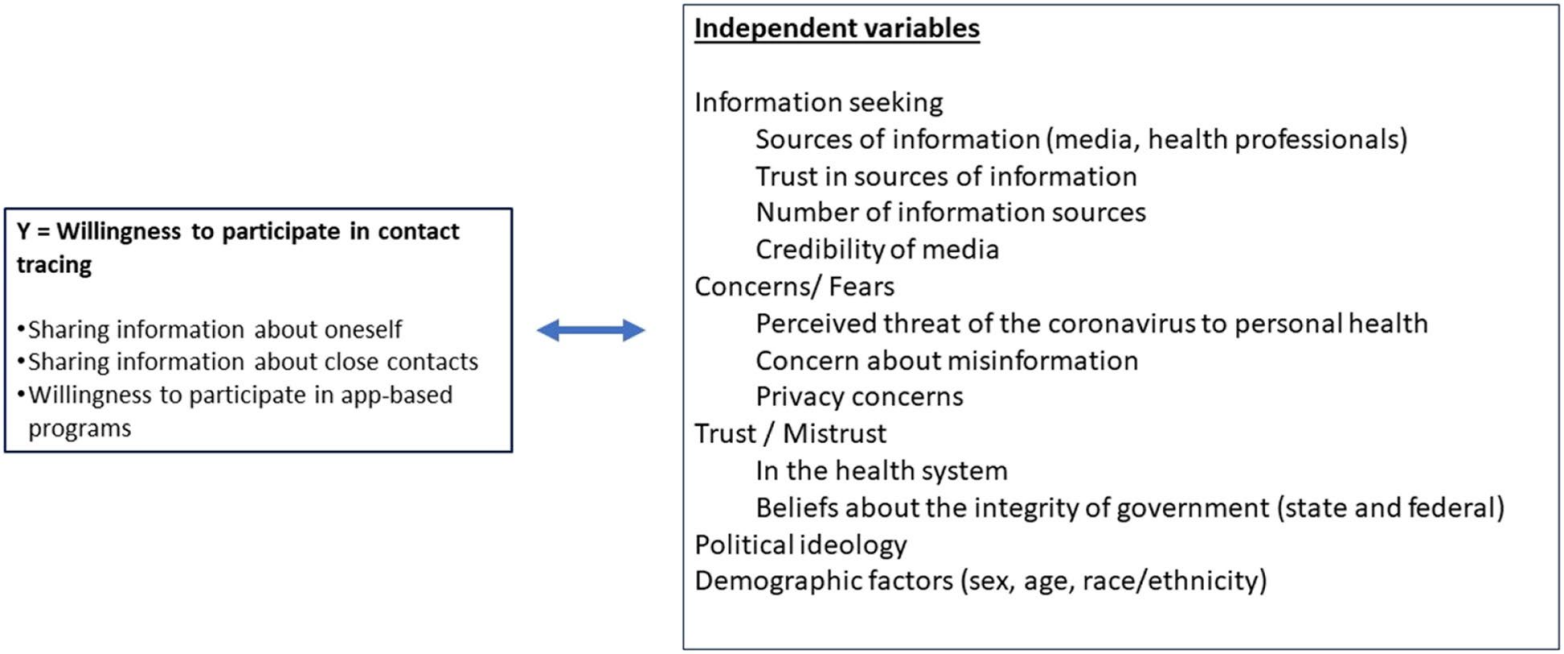
Factors hypothesized to be associated with willingness to participate in contact tracing in the early stages of the COVID-19 pandemic (May 2020)

Information seeking, fear or heightened concerns, trust in the health system and in government, and politics quickly emerged as issues that would shape the public response to the COVID-19 pandemic [8]. We chose to include these factors in our survey design and implementation based on existing theory about health beliefs and behaviors, as described below and shown in Fig. 1. However, we also collected our data early in the pandemic, and before the scientific community had had an opportunity to broadly assess trends and outcomes.

### Information seeking

As the science of the novel coronavirus emerged, the nature of information about COVID-19 also evolved. Conflicting messages from public health professionals and the broadcast media [resulted in confusion and misinformation about the outbreak [9–11] and yet information seeking, including seeking opportunities to discuss the pandemic with trusted experts and social media were one of the primary recourses people had early in the pandemic to reduce uncertainty and mitigate potential harm [12]. In addition to questions about accuracy of information, there were public concerns about the credibility of information—that is, whether the media was accurately communicating the severity of the virus and its potential impact [13, 14].

Information seeking behavior has long been linked to health behaviors by theories such as Eagly and Chaikens (1993) heuristic-systematic model (HSM) of information processing, Ajzen’s (1988) theory of planned behavior, and the Risk Information Seeking and Processing (RISP) model [15, 16]. Each of these point to the connection between information seeking and an individuals’ propensity for following recommended health behaviors to lower one’s risk of preventable disease. We thus sought to capture where people got their information – either via the media or from health professionals –how many outlets individuals consulted, whether those information sources were trusted. We hypothesized that which information sources individuals turned to, and whether these information sources were trusted, would influence attitude formation about mitigation strategies such as contact tracing [15, 16].

### Concerns and fear

In addition to information seeking, fear has a powerful impact on one’s attitudes and clearly shaped early public opinion about the pandemic. Affective response to disease risks such as worry or anxiety affects the desire to seek additional information, influences information processing style and health behavior [17]. In the onset of the pandemic, early adopters of risk reducing behavior such as handwashing and social distancing were also those who perceived the threat of the coronavirus to personal health [18, 19]. Concerns about harm coming from breaches of personal privacy or sharing information with health authorities have been shown to reduce the likelihood that people will be forthcoming with personal information, which is sought during contact tracing [18] .

### Trust and mistrust

Trust and mistrust were also cited as factors contributing to attitudes about the pandemic. These included, for example, public concerns about the integrity of government —that is, whether the government or media was being honest or had a hidden agenda about the virus and its potential impact [9–12, 20]. The spread of misinformation was linked to potential erosion of trust in a variety of information sources [11, 13] and, potentially, willingness to participate in public health interventions such as contact tracing [20].

### Political ideology

The pandemic in the United States came at a time of high political polarization and low levels of trust in government [21]. While some argue that the moral domains of political liberals and conservatives are more likely to shape behavior than party or self-identified political orientation, the novel circumstances in which the U.S. President’s Office directly contradicted guidance from top public health officials made political ideology a central feature to the pandemic. Thus, we hypothesized that political ideology would influence people’s attitudes toward cooperating with government to carry out prevention efforts such as contact tracing [9, 11].

## Methods

### Participants

We utilized publicly available data from the 79th Michigan State of the State quarterly survey (SOSS) conducted in May 2020. The survey was a stratified random sample of 1086 non-institutionalized, English-speaking Michigan adults who could be reached by cell phone or landline. About 35% of the sample of interviews were derived from re-contacts. The remaining 65% of the sample was derived from a random-digit-dial sample of phone numbers in the state. Non-response adjustments were made to ensure the sample was representative of the state’s adult population. The sample was matched to a sampling frame of 1000 respondents, constructed from the 2016 American Community Survey. Matching was based on gender, age, race, and education and weighted to the sampling frame using propensity scores [22, 23].

### Data collection

Interviews were conducted using the computer assisted telephone interviewing system (CATI) of the Institute for Public Policy and Social Research’s (IPPSR) Office of Survey Research (OSR) [22, 23]. CATI is a survey modality in which interviewers follow a script on the computer to conduct interviews by telephone. The OSR CATI system uses built in logic that allows for sequential movement from one question to the other and automatic skip patterns depending on responses [23]. The dataset analyzed during the current study is available on the Michigan State University website at, http://ippsr.msu.edu/survey-research/state-state-survey-soss/soss-data/soss-79b-spring-2020.

### Dependent variable

The aim of this study was to assess the association between information seeking, concerns/fear about the pandemic, trust, and political ideology and willingness to participate in contact tracing in Michigan.

#### Factors contributing to willingness to participate in contact tracing

Given the importance of contact tracing to public health efforts in mitigating the spread of COVID-19, we examined the factors associated with willingness to participate in contact tracing. We drew on the literature about the use of information technologies such as apps, preventative health behaviors, and the context of COVID-19 at the time.

Our main outcome variable was a composite measure of willingness to participate in contact tracing efforts derived from responses to three questions. Responses were measured on a seven-point scale ranging from “Not true at all” [1] to “Very true” [7]. The composite index for each respondent was the average measure of the following three questions: (i) “I would feel comfortable reporting people I’ve been in contact with to the local or state health department if I had symptoms of COVID-19” (ii) “I would be comfortable using a computer or phone app that shares my symptom information with my local or state health department” and (iii) “I am willing to give my local or state health department personal information to help limit the spread of COVID 19” (Cronbach’s Alpha: 0.87 (CI = 0.85, 0.88).

### Independent variables: predictors of willingness to participate in contact tracing

We included variables capturing the following factors potentially associated with willingness to participate in contact tracing: (1) information seeking, (2) concerns and fears, (3) trust/ mistrust, (4) political ideology, and (5) demographic factors (Tables 1 & 2). Unless otherwise noted, responses were measured on a seven-point scale assessing “How True” people felt a series of statements were. Responses ranged from “Not true at all” [1] to “Very true” [7].

**Table 1:** Estimates of internal consistency of combined variables assessing willingness to participate in contact tracing, and information seeking behavior (*N* = 1000)

	Questions	Median (IQR)^a^	Overall Cronbach’s alpha (95% CI)^b^
**Willingness to participate in contact tracing**	**Comfort with and willingness to participate in contact tracing (Range: 1 = Not true at all; 7 = Very true)**	5 (7–3.7)	**0.87 (0.85, 0.88)**
	*For you, how true are the following statements* **(Range: 1 = Not true at all; 7 = Very true)**		
	I would feel comfortable reporting people I have been in contact with to the local or state health department if I had symptoms of the coronavirus	6 (7–4)	
	I would be comfortable using a computer or phone app that shares my symptom information with my local or state health department	5 (6–3)	
	I am willing to give my local or state health department personal information to help limit the spread of the coronavirus	5 (6–4)	
**Information seeking**	*Thinking about some of the ways you get information about the coronavirus outbreak, would you say that you get information from each of the following sources?*		
	**Get information from public health institutions (Range: 1 = Never; 4 = Regularly)**	2 (2.3–1.3)	**0.68 (0.65, 0.72)**
	Centers for Disease Control (CDC)	2 (3–1)	
	Michigan Department of Health and Human Services	2 (3–1)	
	County Health Department	1 (2–1)	
**Information seeking**	**Get information from national left leaning media sources**^**c**^ **(Range: 1 = Never; 4 = Regularly)**	1 (1.5–1)	**0.78 (0.76, 0.80)**
	CBS News	1 (2–1)	
	MSNBC	1 (2–1)	
	ABC News	1 (2–1)	
	New York Times	1 (2–1)	
	The Daily Show or Colbert Report	1 (2–1)	
	The Washington Post	1 (2–1)	
**Information seeking**	**Get information from national right leaning media sources**^**c**^ **(Range: 1 = Never; 4 = Regularly)**	1 (1.3–1)	0.61 (0.56, 0.65)
	Fox News	1 (2–1)	
	Rush Limbaugh Show	1 (1–1)	
**Information seeking**	**Get information from local media sources**^**c**^ **(Range: 1 = Never; 4 = Regularly)**	1 (1.5–1)	**0.75 (0.72, 0.77)**
	Detroit Free Press	1 (2–1)	
	Detroit News	1 (1–1)	
	MLive	1 (1–2)	
	Lansing State Journal	1 (1–1)	
**Information seeking**	*Regardless of how often you get information from these sources, how much do you trust information provided about the coronavirus outbreak by each of the following?*		
	**Trust information from public health institutions**^**c**^ **(Range: 1 = Not at all; 5 = A great deal)**	3.7 (4.3–2.7)	**0.85 (0.84, 0.87)**
	Centers for Disease Control (CDC)	4 (5–3)	
	Michigan Department of Health and Human Services	4 (5–3)	
	County Health Department	3 (4–2)	
**Information seeking**	**Trust information from national left-centralist media sources**^**c**^ **(Range: 1 = Not at all; 5 = A great deal)**	2.5 (3.5–1.3)	**0.93 (0.92, 0.94)**
	CBS News	3 (4–1)	
	MSNBC	2 (4–1)	
	ABC News	3 (4–1)	
	New York Times	3 (4–1)	
	The Daily Show or Colbert Report	2 (3–1)	
	The Washington Post	2 (4–1)	
**Information seeking**	**Trust information from national right leaning media sources**^**c**^ **(Range: 1 = Not at all; 5 = A great deal)**	1.5 (3–1)	**0.77 (0.74, 0.80)**
	Fox News	2 (3–1)	
	Rush Limbaugh Show	1 (2–1)	
**Information seeking**	**Trust information from local media sources**^**c**^ **(Range: 1 = Not at all; 5 = A great deal)**	2.3 (3–1.3)	**0.91 (0.90, 0.92)**
	Detroit Free Press	2.5 (3–1)	
	Detroit News	2 (3–1)	
	MLive	2 (3–1)	
	Lansing State Journal	2 (3–1)	
**Information seeking**	**Median number of information sources per respondent**	**8 (11–5)**	
**Trust/ Mistrust**	**General trust in health system (Range: 1 = Not true at all; 7 = Very true)**	5.3 (6–4.3)	**0.87 (0.85, 0.88)**
	***For you, how true are the following statements?***		
	All things considered, health care providers in this country can be trusted	5 (6–4)	
	The organization where I typically get health care can be trusted to use my information responsibly	5 (6–4)	
	The organization where I typically get health care protects my privacy	6 (6–4)	

^a^IQR means Interquartile range. ^b^CI Means confidence interval. ^c^ Media sources classified according to political ideology spectrum described by the Pew Research Foundation: Pew Research Center. Political polarization and media habits. 2014, October 24. Retrieved from https://www.pewresearch.org/journalism/2014/10/21/political-polarization-media-habits/pj_14-10-21_mediapolarization-08/

**Table 2:** Descriptive statistics and bivariate association between potential predictors and willingness to participate in contact tracing [*N* = 1000] (Categorical variables)

Characteristic	Frequency (%)		Mann Whitney U/ Kruskal Wallis tests^b^			
		Median (IQR)^a^	U/H-Statistics	df^c^	z	*p*-value
**Demographics**						
**Gender**			206,062	1	−1.478	0.139
Males	48.0%	4.3 (6.0–3.0)				
Female	52.0%	4.7 (6.0–3.7)				
**Race/Ethnicity**			95,173	1	2.797	0.005
White	83.7%	4.3 (6.0–3.0)				
Black	16.3%	5.0 (6.0–4.0)				
**Education**			9.26	2	–	0.010
College graduate or higher	30.0%	5.0 (6.0–3.7)				
Some college	29.7%	4.7 (6.0–3.3)				
High school graduate or less	40.3%	4.0 (6.0–3.0)				
**Information seeking**						
**Thinking about what is said in the news, in your view is the seriousness of coronavirus….?**			237.01	2		<.0001
Generally, correct	43.8%	5.0 (6.3–4.0)				
Generally exaggerated	34.4%	3.0 (4.3–1.0)				
Generally underestimated	21.8%	5.3 (6.7–4.0)				
**Thinking about some of the ways you get information about the coronavirus outbreak, would you say that you get information from each of the following sources… [Range 1 = Never; 4 = Regularly]**						
**Health care providers**			11.1591	2	–	0.004
Never, or rarely	61.7%	4.3 (6.0–3.0)				
Occasionally	27.8%	5.0 (6.0–4.0)				
Regularly	10.5%	5.0 (6.0–3.0)				
**Social media**			5.9814	2	–	0.050
Never, or rarely	47.9%	4.3 (6.0–3.3)				
Occasionally	32.0%	4.3 (6.0–3.0)				
Regularly	20.1%	5.0 (6.3–4.0)				
**Health insurance provider**			11.1662	2	–	0.004
Never, or rarely	71.4%	4.3 (6.0–3.7)				
Occasionally	21.5%	5.0 (6.0–3.7)				
Regularly	7.0%	5.3 (6.3–4.0)				
**Regardless of how often you get information from these sources, how much do you trust information provided about the coronavirus outbreak by each of the following? [Range 1 = Not at all, 7 = a great deal]**						
**Healthcare providers?**			92.4959	2	–	<.0001
Not at all, or only a little	18.8%	4.0 (4.7–2.0)				
A moderate amount	23.1%	4.0 (6.0–3.3)				
Quite a bit or a great deal	58.1%	5.3 (6.3–3.7)				
**Family and Friends**			13.3238	2	–	0.0013
Not at all, or only a little	28.7%	4.0 (5.3–3.0)				
A moderate amount	41.1%	5.0 (6.0–3.7)				
Quite a bit or a great deal	30.2%	4.7 (6.3–3.3)				
**Insurance providers**			89.8981	2	–	<.0001
Not at all, or only a little	36.0%	4.0 (5.3–2.0)				
A moderate amount	34.8%	4.7 (6.0–3.3)				
Quite a bit or a great deal	29.2%	5.7 (6.7–4.3)				
**Trust/ Mistrust**						
**I think the federal government has an agenda that’s causing them not to give the whole story to the public. [Range 1 = Not true at all, 7 = Very true]**			46.20	2	–	<.0001
Not true at all, somewhat untrue or untrue	19.9%	4.0 (5.7–2.0)				
Neutral	19.3%	4.0 (5.3–3.7)				
Somewhat True, true, or very true	60.8%	5.0 (6.3–3.7)				
**I think the Governor’s office has an agenda that’s causing them not to give the whole story to the public.** **.... [Range 1 = Not true at all, 7 = Very true]**			206.42	2	–	<.0001
Not true at all, somewhat untrue or untrue	39.5%	6.0 (6.7–4.7)				
Neutral	14.2%	4.0 (5.3–3.7)				
Somewhat True, true, or very true	46.3%	4.0 (5.0–2.0)				
**Fears/ Concerns**						
**I worry that private information about my health could be used against me**			71.33	2	–	<.0001
Not true at all, somewhat untrue or untrue	29.5%	5.7 (6.7–4.0)				
Neutral	25.0%	4.3 (5.7–4.0)				
Somewhat True, true, or very true	45.5%	4.0 (5.7–2.7)				
**I am worried that misinformation about COVID 19 is making people less safe? [Range 1 = Not true at all, 7 = Very true]**			75.77	2	–	<.0001
Not true at all, somewhat untrue or untrue	13.5%	3.3 (5.0–1.0)				
Neutral	12.2%	4.0 (4.3–3.3)				
Somewhat True, true, or very true	74.3%	5.0 (6.3–3.7)				
**How much of a threat is the coronavirus outbreak to your personal health?**			141.87	2	–	<.0001
No threat/Don’t know	25.2%	3.3 (4.7–1.3)				
Minor threat	44.0%	4.7 (6.0–3.7)				
Major threat	30.8%	6.0 (6.7–4.3)				
**Political Ideology**						
**Do you think of yourself as a….?**			173.28	2	–	<.0001
Moderate or Other	41.1%	4.6 (6.0–3.7)				
Conservative	28.7%	3.7 (4.7–1.7)				
Liberal	30.2%	6.0 (6.7–4.3)				

^a^IQR means interquartile range. ^b^Mann Whitney U also called Wilcoxon rank sum test was used for categorical variables with two levels while Kruskal Wallis test was used for categorical variables with more than two levels. df means degrees of freedom

*Information seeking* was captured by questions about where people got information about COVID-19, trusted sources of information, frequency of information seeking, number of sources and whether they thought the media fairly portrayed the seriousness of the pandemic.

Survey respondents were asked to indicate frequency (regularly, occasionally, rarely, never) and level of trust (Not at all (1) – A great deal (7)) in information sources including public health institutions (CDC, state, and local health departments), national media sources, local media sources, healthcare (health care providers, insurance companies), and social networks (friends and family). We aggregated responses about national media sources to capture frequency and trust in right and left centrist sources, categorized as such based on a spectrum defined by the Pew Research Center [24]. The full set of questions is provided in Table 1.

To assess the total number of information sources, we created dummy variables equal to 1 if the respondent reported getting information occasionally or regularly from an information source and zero otherwise and summed across all 21 sources to compute the total number of sources of information on COVID-19 for each respondent. Table 1 summarizes variables generated from multiple questions and their internal consistency (Cronbach’s alpha).

We asked three questions about various *concerns and fears* related to the pandemic. First, we asked about the perceived threat of the coronavirus to personal health. Second, to assess concern about the harmful effect of misinformation we asked the extent to which the statement, “I am worried that misinformation about COVID-19 is making people less safe” was true or not true. Third, we asked whether they were concerned about private information being used against them.

*Trust and mistrust* were measured using a composite measure of general trust in the health system based on three questions (Cronbach’s alpha: 0.87, 95% CI: 0.85, 0.88) (see Table 1). To assess mistrust, i.e., beliefs about the integrity of state and federal government, we asked people to indicate how true they felt two statements to be: “I think the Governor’s office has an agenda that’s causing them not to give the whole story to the public,” and “I think the federal government has an agenda that’s causing them not to give the whole story to the public” (Emphasis added).

*Political ideology* [conservative, moderate, liberal, or other] was also measured given the politicized nature of the pandemic throughout its development. Respondents reported *demographic factors*, age, sex, race/ethnicity [White, Black, Other (not reported)], and education [less than high school, high school graduate, some college, college graduate or higher], which we included in our statistical analysis.

### Statistical analysis

The variables analyzed were ordinal variables with non-normal distributions. Quantile-quantile (QQ) plots of all variables appeared linear, but the distributions of the variables were negatively or positively skewed [25]. The Shapiro-Wilk and Anderson Darling normality tests all resulted in *p*-values that were less than 0.05, implying that the assumptions of normality were not satisfied. Spearman rank-order correlation and Mann Whitney U tests were used to analyze the data. Both methods are non-parametric methods that do not rest on the assumption of normality and are suitable for the analysis of continuous and ordinal data.

The data contained 12% missing data, which were imputed using hot deck imputation. Hot deck imputation is appropriate when missingness is random [26], which we assessed by visual inspection. Visual inspection showed arbitrary or unstructured missing data patterns with no evident mechanism, suggesting that missingness was ignorable.

Spearman rank-order correlation analysis was conducted to determine the strength and direction of the association between the dependent variable (willingness to participate in contact tracing) and each continuous independent variable (Table 3). We also conducted exploratory bivariate analysis to examine the relationship between the dependent variable and each categorical independent variable (Table 2). Dependent and independent variables used in regression analysis were standardized to allow for simple comparisons of effect sizes and to facilitate interpretation.

**Table 3:** Association between willingness to participate in contact tracing and information seeking, trust, and demographic characteristics of survey respondents (*n* = 1000) (Continuous Variables)

	Variable	Median (IQR)^a^	Spearman correlation with contact tracing		
			R	(95%, CI)^b^	*p*-value
**Dependent variable**	**Willingness to participate in contact tracing**	4.7 (6.0–3.3)	NA	NA	NA
**Demographics**	Age	51 (64–35)	0.04	− 0.018, 0.105	0.1669
**Information seeking**	**Thinking about some of the ways you get information about the coronavirus outbreak, would you say that you get information from each of the following sources… [Range 1 = Never; 4 = Regularly] (Information seeking)**				
	Public health institutions	1.7 (2.3–1.3)	0.40	0.343, 0.447	<.0001
	National left leaning media sources	1.3 (1.7–1.0)	0.44	0.391, 0.492	<.0001
	National right leaning media sources	1.0 (1.5–1.0)	−0.27	−0.330, − 0.216	<.0001
	Local news sources	1.0 (1.5–1.0)	0.24	0.177, 0.294	<.0001
	**Number of information sources**	7.0 (10.0–5.0)	0.34	0.282, 0.392	<.0001
	**Regardless of how often you get information from these sources, how much do you trust information provided about the coronavirus outbreak by each of the following? [Range 1 = Not at all, 7 = a great deal]**				
	Public health institutions	3.3 (4.0–2.3)	0.53	0.479, 0.569	<.0001
	National left leaning media sources	2.2 (3.3–1.2)	0.54	0.499, 0.587	<.0001
	National right leaning media sources	2.0 (3.0–1.0)	−0.32	−0.317, − 0.259	<.0001
	Local sources	2.3 (3.0–1.3)	0.42	0.372, 0.474	<.0001
**Trust**	**General trust in health system**	5.0 (6.0–4.0)	0.45	0.402, 0.500	<.0001

^a^IQR means interquartile range. ^b^CI Means confidence interval

Building our final model followed a structured approach. Based on the exploratory bivariate analysis (Mann Whitney and Spearman rank correlations), we subjected candidate predictors with significant relationships (*p* < 0.10) with the outcome variable to stepwise regression. Stepwise regression utilized *p* < 0.05 as inclusion criteria with the selection process terminating when adding any variable to the model increased the predicted residual sum of squares.

Based on the results of the stepwise regression, we ran a multiple linear regression model to investigate the relationship between willingness to participate in contact tracing and independent variables identified in the stepwise regression. Analysis of residuals was used to check for model assumptions. The assumptions of normality of residuals and homogeneity of variance were not violated. There were few potential outliers, but Cook’s distance values computed to assess the effect of the outliers were less than 0.28, below the influential point threshold of one or higher [27]. A test for multicollinearity showed that variance inflation factors were within acceptable limits ranging from 1.1 to 2.6 [28], indicating that there was no substantial multicollinearity in the model [29]. Thus, the final model fits the data.

## Results

### Sample demographics

The sample was predominantly White (83.7%), with slightly more females (52.0%) than males. The mean age of respondents was 48.0 years (SD = 17.9). Two-fifths of the respondents had a high school education or lower (40.3%), while 29.7% had some college education and 30.0% had a graduate degree or higher (30.0%). Approximately 41.1% of respondents identified as politically moderate, 28.7% identified as conservative and 30.2% identified as liberal (Table 2).

### Dependent variable: willingness to participate in contact tracing

Over half of the survey respondents reported that they would be comfortable sharing personal information with their local public health department (52.9%), sharing contacts (58.8%), and reporting symptoms to local or state health departments (46.6%). The composite measure of these three questions assessing willingness to participate in contact tracing (Mdn = 5, IQR = 7–3.7) showed good internal consistency (Cronbach’s α = .87 with 95% confidence interval (CI) of 0.85 to 0.88).

### Independent variables and their relationship to willingness to participate in contact tracing

#### Information seeking

Just over one-third of the respondents (34.4%) felt that what was said in the news about the coronavirus was exaggerated, 21.8% said it was underestimated and 43.8% felt it was about correct. Approximately 81.2% of the respondents trusted information about the coronavirus from personal health care providers, but only 38.3% reported getting most of their information about COVID-19 from their health care providers. Similarly, 71.9% said public health institutions were trusted sources of information, while 58.0% of respondents got information from these institutions.

The Spearman rank-order correlation tests at the 95% confidence interval (CI) showed that getting information from right leaning media sources was negatively correlated with getting information from public health institutions, [r (998) = − 0.17, *p* < .001, CI = − 0.23, − 0.11] and trust in information from these institutions, [r (998) = − 0.33, *p* < .001, CI = -0.38, − 0.27]. Conversely, getting information from left leaning media sources was positively associated with getting information about COVID-19 from public health institution [r (998) =0.49, *p* < .001, CI = 0.45, 0.54) and trust in information about COVID-19 from these sources [r (998) = 0.49, *p* < .001, CI = 0.44, 0.53).

Bivariate analysis was also conducted to examine the association between various categorical variables and willingness to participate in contact tracing. Mann Whitney test was used for categorical variables with two levels while Kruskal Wallis test was used for categorical variables with more than two levels (Table 2). The results showed that the median willingness to participate in contract tracing differed by whether participants rarely, occasionally, or regularly got or trusted information from various sources, and whether they perceived that what was said in the news about the pandemic was correct, exaggerated or underestimated (Table 2). However, median willingness to participate in contact tracing did not differ, at the 95% confidence level, by whether participants never, occasionally, or regularly got information from social media sources.

#### Trust/ mistrust

Approximately 61.0% of respondents reported that health care providers in the US can be trusted. About 64.5 and 67.2% expressed trust in health care providers to use personal information responsibly and to protect privacy respectively. As a combined measure, general trust in the health system was shown to be positively associated with willingness to participate in contact tracing (*p* < 0.001). Approximately three out of five respondents (60.8%) said it was true that the Federal Government had “a hidden agenda causing them not to tell the whole story about the coronavirus” compared to two out of five (46.3%) who said the Michigan Governor’s Office was not telling the whole story about the disease. In unadjusted analysis, participation in contact tracing differed by trust or mistrust of the Federal and State Governments’ handling of the pandemic (Table 2).

#### Concerns/ fears

Approximately 45.5% of the respondents were somewhat or very worried that private information provided for the purpose of health care could be used against them. About 74.3% of respondents expressed concern about the harmful effects of misinformation. Three out of ten (30.8%) respondents perceived the coronavirus pandemic to be a major threat to personal health while 25.2% felt it was no threat. Median score on willingness to participate in contact tracing differed by levels of fear or concern about misinformation and misuse of private information (Table 2). Similarly, median score on willingness to participate in contract tracing differed by the extent to which the participants felt COVID-19 was a major, minor or no threat to personal health (i.e., perceived risk was associated with contact tracing).

#### Political ideology

About 30.2% of the respondents were liberal, 28.7% were conservative, while 41.1 were moderates or hold another political ideology. The results of a Kruskal Wallis test showed that the willingness to participate in contact tracing differ by political ideology (i.e., liberal, conservative, or moderate).

#### Demographic factors

In unadjusted analysis, median willingness to participate in contact tracing did not differ by gender or age but differed by educational level and race/ethnicity (Table 2).

Table 3 shows the results of the bivariate analysis of the association between various information sources and willingness to participate in contact tracing. The moderate to high Spearman correlation coefficients indicate that the sources of information were important in determining whether Michigan adults participated in the state’s contact tracing efforts.

### Factors associated with willingness to participate in contact tracing: stepwise regression

The multiple linear regression included variables that had a significance level of *p* < 0.05 in the stepwise regression. However, gender and age were retained in the model. The final model is summarized in Table 4.

**Table 4:** Multivariable model identifying predictors of willingness to participate in contact tracing based on stepwise regression [*N* = 1000]

	Multivariable Standardized Regression Estimates R^2^ = 0.48 (0.43, 0.51); F = 38.82; df^a^ = 20, *p* < .0001			
Variable	Estimates	*p*-value	95% Confidence Limits	
			Lower	Upper
**Demographics**				
**Sex**				
Male	ref.		ref.	
Female	0.01	0.882	−0.09	0.10
**Race/Ethnicity**				
White	ref.		ref.	
Black	0.20	0.004	0.07	0.33
**Information seeking**				
**Thinking about some of the ways you get information about the coronavirus outbreak, would you say that you get information from each of the following sources…? [Range 1 = Never; 4 = Regularly]**				
**Health care providers**	ref.		ref.	
Never, or rarely				
Occasionally	0.07	0.256	−0.05	0.19
Regularly	−0.11	0.189	−0.28	0.06
**Public health institutions**^**b**^	0.10	0.013	0.02	0.17
**Trusted sources of information**				
**Regardless of how often you get information from these sources, how much do you trust information provided about the coronavirus outbreak by each of the following sources…? [Range 1 = Not at all, 7 = a great deal]**				
**Family and Friends**	ref.		ref.	
Not at all, or only a little				
A moderate amount	0.10	0.096	−0.02	0.22
Quite a bit or a great deal	−0.09	0.177	−0.22	0.04
**Public health institutions**^**c**^	0.10	0.006	0.03	0.18
**National left leaning media sources**^**d**^	0.17	<.001	0.09	0.24
**National right leaning media sources**^**e**^	−0.08	0.012	−0.14	−0.02
**Number of information sources**	0.01	0.11	0.00	0.03
**Concerns/ fears**				
**I am worried that misinformation about COVID 19 is making people less safe? [Range 1 = Not true at all, 7 = Very true]**				
Not true at all, somewhat untrue or untrue	ref.		ref.	
Neutral	0.19	0.049	0.00	0.39
Somewhat True, true, or very true	0.30	<.001	0.16	0.45
**How much of a threat is the coronavirus outbreak to your personal health?**				
No threat/Don’t know	ref.		ref.	
Minor threat	0.33	<.001	0.20	0.45
Major threat	0.59	<.001	0.45	0.73
**I worry that private information about my health could be used against me**				
Not true at all, somewhat untrue or untrue	ref.		ref.	
Neutral	−0.11	0.111	−0.25	0.03
Somewhat True, true, or very true	−0.35	<.001	−0.47	− 0.23
**Trust**				
**General trust in health system**	0.17	<.001	0.12	0.23
**Political Ideology**				
**Do you think of yourself as a….?**				
Moderate or Other	ref.		ref.	
Conservative	−0.12	0.076	−0.25	0.01
Liberal	0.06	0.318	−0.06	0.19

^a^ *df* degrees of freedom. ^b^Get information from public health institutions (CDC, State Health Department & County Health Department). ^c^Trust information from Public Health Institutions (CDC, State Health Department & County Health Department). ^d^Trust information from national left leaning media sources (CBS News, MSNBC, New York Times, The Daily Show, ABC News, and The Washington Post). ^e^Trust information from national right leaning media sources (Fox News, and Rush Limbaugh Show)

A detectable overall association was found, (F (20, 999) =38.82, *p* < 0.001. The coefficient of determination (R^2^) was 0.48 with confidence interval of 0.43 to 0.51, indicating that 48% of the variance in willingness to participate in contact tracing was explained by the predictor variables.

The results show that a one standard deviation increase in concern about the harmful effect of misinformation resulted in a 0.30 increase in willingness to participate in contact tracing in adjusted analysis (*p* = 0.001; Ref = Not true at all, somewhat untrue or untrue). After adjusting for other covariates, the perception that COVID-19 was a major threat (B = 0.59, *p* < 0.001) or minor threat (B = 0.33, *p* = 0.001; Ref = No threat) to personal health was the strongest positive predictor of comfort with and willingness to participate in contact tracing. Trust in information from left leaning media sources (B = 0.17, *p* < 0.001), and general health system trust (B = 0.17, *p* < 0.001) were also positively associated with contact tracing. Conversely, trust in information from right leaning media sources (B = -0.08, *p* = 0.012) was marginally and negatively associated with contact tracing. Concerns about the misuse of personal health information to the detriment of the respondent (B = -0.35, *p* < 0.001; Ref = Not true at all, somewhat untrue or untrue) had the highest negative association with contact tracing.

## Discussion

This study explored factors associated with willingness to participate in contact tracing during the COVID-19 pandemic in the State of Michigan: information seeking, concerns and fears, trust and mistrust, and political ideology.

Previous research on information seeking and attitudes such as fear and trust about a risk, such as infection with COVID-19 or other diseases, suggests that beliefs about the quality of information available on the health risk from various sources is linked to how much effort people will expend to seek and critically analyze information from these sources [15, 16]. Trusted sources that effectively conveyed the seriousness of the threat and provided information about how individuals could control the danger improved the adoption of recommended behavior [18, 30].

We represented information seeking behavior by the participants’ number, type, and trusted sources of information about COVID-19. We found that getting and trusting information from public health institutions and trusting information from media sources, were associated with willingness to participate in contact tracing, with trust in liberal sources being positively and conservative media sources negatively associated with willingness to participate in contact tracing. These findings are consistent with evidence from studies that have shown that during a crisis, information from trusted institutional sources can help shape accurate public perception of the risk, perceived vulnerability, and fear of personal safety, which are associated with intention to adopt appropriate public health behavior [31].

In the case of the COVID-19 pandemic, our study suggests that different media platforms shaped different public responses – i.e., those who sought information from politically conservative media sources were less likely to be willing to participate in contact tracing. Our findings also suggest that the political climate of the U.S. at the outset of the pandemic permeated the media, the nature of trusted media sources, and willingness to participate in contact tracing. Specifically, the association between getting information about COVID-19 from trusted media sources and willingness to participate in contact tracing varied depending on the political lens of the media source. We found that trusting information about COVID-19 from national left leaning media sources (MSNBC, ABC News, CBS News, New York Times, & Washington Post), was positively associated with willingness to participate in contact tracing. Contrarily, trusting information from right leaning sources (Fox News, and Rush Limbaugh Show) was negatively associated with willingness to participate in contact tracing. Previous studies have reported that right leaning media sources were a source of COVID-19 related misinformation, mirroring the position of Republican leadership, which downplayed the need for government interventions to curtail the spread of the virus [11, 13, 14]. One study that examined the causal effect of Fox-News viewership on compliance with recommendations by health experts found that “a 10% increase in Fox News viewership led to a 1.3%-point reduction in propensity to stay at home” [14]. Our results exemplify the reported partisan differences between Democrats (mostly identified as liberals) and Republicans (mostly identified as conservatives) about the appropriate policy response to the pandemic [9–11].

We also found that common sources of information were not necessarily also trusted sources of information. For example, while most people trusted information about COVID-19 from their health care providers, this was not the most frequently cited source of information, and most people got their information instead from various media. This underscores the importance of increasing the availability and accessibility of, for example, care providers and public health institutions to people in the early stages of a public health crisis.

The communication approaches of these trusted leaders and organizations will need to manage not only emerging and new information, but also the concerns and fears of the public. In our study, fears, and concerns about threats to personal health and community well-being predicted positive willingness to participate in contact tracing, while concerns about privacy negatively predicted willingness to participate in contact tracing. Media and other information sources, then, need to be mindful of how fear-based messages might impact attitudes about public health programs. Notably, general trust in health system was positively associated with willingness to participate in contact tracing, suggesting the need to focus on efforts to build or re-build trust as COVID-19 approaches endemicity and as a part of preparedness for new public health emergencies.

### Limitations

Our study has limitations. We conducted a cross-sectional study, so any correlations identified cannot suggest causation. Future studies should engage in longitudinal designs to establish causation. We also did not include interaction terms, which may account for additional relationships between our variables beyond those tested in our models. The sample was limited to the Michigan population who lived in households that had landline telephones or individuals who had a cell phone. Thus, the data is subject to coverage errors. To address this challenge, the sample was appropriately stratified and weighted to account for disproportionate selection probabilities. Nevertheless, the external validity of our study findings is limited to the non-institutionalized, English-speaking adult population of Michigan age 18 and over.

## Conclusion

The strongest positive predictors of comfort with and willingness to participate in contact tracing were the perceived threat of the pandemic to personal health, worry about the harmful effects of misinformation and general trust in health system. Concerns about privacy of information provided to public health institutions was the strongest negative predictor of willingness to participate in contact tracing. Helping the public appreciate the seriousness of the pandemic in ways that increase transparency and demonstrate the trustworthiness of information and institutions, addressing concerns about potential harms, and communicating accurate information using trusted sources across political ideologies could improve willingness to participate in contact tracing, and public health programs more generally. Our findings indicate that healthcare providers and public health institutions were highly trusted sources of information about COVID-19 but were not the main sources of information, suggesting that raising their visibility as regular sources of information may be an important strategy in engaging the public.

## Data Availability

This manuscript contain all the evidence that support the findings and conclusions. All methods were carried out in accordance with relevant guidelines and regulations. However, the data that support the findings of this study is publicly available and can be accessed at http://ippsr.msu.edu/survey-research/state-state-survey-soss/soss-data. Please direct request or comments to Matt Grossmann, Director of the State of the State Survey at grossm63@msu.edu
